# Composing the Early Embryonic Microenvironment: Physiology and Regulation of Oviductal Secretions

**DOI:** 10.3390/ijms21010223

**Published:** 2019-12-28

**Authors:** Marie Saint-Dizier, Jennifer Schoen, Shuai Chen, Charles Banliat, Pascal Mermillod

**Affiliations:** 1 Faculty of Sciences and Techniques, Department Agrosciences, University of Tours, 37200 Tours, France; 2 Institut National de la Recherche Agronomique (INRA), UMR85 Physiologie de la Reproduction et des Comportements, CNRS 7247, University of Tours, IFCE, 37380 Nouzilly, France; charles.banliat@inra.fr (C.B.); pascal.mermillod@inra.fr (P.M.); 3 Leibniz Institute for Farm Animal Biology, FBN Dummerstorf, 18196 Dummerstorf, Germany; schoen.jennifer@fbn-dummerstorf.de (J.S.); chen@fbn-dummerstorf.de (S.C.); 4 Union Evolution, Rue Eric Tabarly, 35538 Noyal-Sur-Vilaine, France

**Keywords:** oviduct, fallopian tube, tubal fluid, female genital tract, cattle, pig, embryo development

## Abstract

The oviductal fluid is the first environment experienced by mammalian embryos at the very beginning of life. However, it has long been believed that the oviductal environment was not essential for proper embryonic development. Successful establishment of in vitro embryo production techniques (which completely bypass the oviduct) have reinforced this idea. Yet, it became evident that in vitro produced embryos differ markedly from their in vivo counterparts, and these differences are associated with lower pregnancy outcomes and more health issues after birth. Nowadays, researchers consider the oviduct as the most suitable microenvironment for early embryonic development and a substantial effort is made to understand its dynamic, species-specific functions. In this review, we touch on the origin and molecular components of the oviductal fluid in mammals, where recent progress has been made thanks to the wider use of mass spectrometry techniques. Some of the factors and processes known to regulate oviductal secretions, including the embryo itself, as well as ovulation, insemination, endogenous and exogenous hormones, and metabolic and heat stress, are summarized. Special emphasis is laid on farm animals because, owing to the availability of sample material and the economic importance of fertility in livestock husbandry, a large part of the work on this topic has been carried out in domestic animals used for dairy and/or meat production.

## 1. Introduction

**Figure 1:**
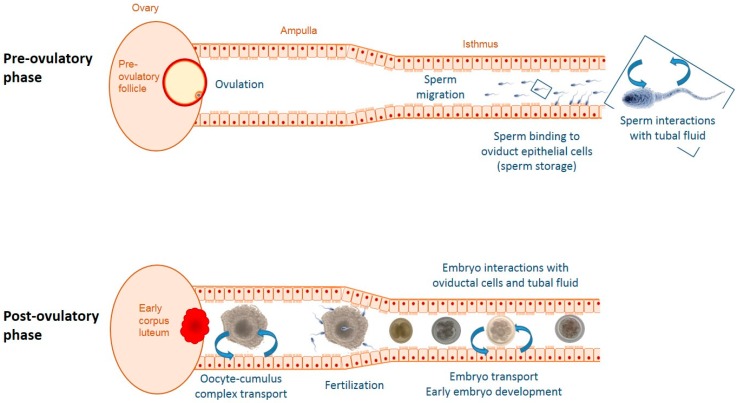
Schematic representation of some factors and processes responsible for changes in the oviductal microenvironment before and during embryonic development. Prior to ovulation sperm interact with the epithelial lining of the oviduct. After ovulation, the ovulated oocyte with surrounding cumulus cells as well as the developing embryo influence oviductal functions both mechanically and via para-/juxtacrine signaling. Throughout the cycle, ovarian sex steroids produced by the follicle(s) and corpora lutea modulate oviductal secretions.

The oviduct has long been considered as a pipeline for the meeting of gametes at ovulation and transport of the embryo to the uterus. From the late 1970s, it became possible to bypass the oviduct and produce mammalian embryos in vitro, leading to the idea that only minimal components of the oviduct were necessary to support embryo development. However, since then, a number of studies showed that the morphology, gene expression, and lipid and protein composition of the in vitro produced embryos differ markedly from their in vivo counterparts, and that these differences are associated with lower pregnancy outcomes and more health issues after birth [1,2,3,4]. Forty years after the first in vitro fertilization (IVF) success and in parallel with limited progress in the field, researchers consider the oviduct as the most suitable microenvironment for early embryo development and substantial effort has been made to understand its dynamic species-specific functions. The oviduct is indeed the first interface between the female genital tract and the conceptus, mediating an early embryo–maternal dialog through cell contacts, secreted molecules, and extracellular vesicles (EVs) [5,6,7]. However, studying the physiology of the oviduct and its secretions to better understand the needs of the embryo is challenging. First, the oviducts are small intra-abdominal organs, not accessible without surgery or animal slaughter and filled with only microliters of fluid. Moreover, many steps in a relatively short period of time take place before embryo development in the oviduct, that is, sperm storage, sperm transport and capacitation, uptake and transport of the cumulus–oocyte complex (COC), and finally fertilization, with each step being able to modify the embryonic microenvironment (Figure 1). Beside these internal factors, various factors outside the oviduct are susceptible to regulate the oviduct microenvironment, including metabolic and environmental stress. The aim of this review is to provide recent data on the molecular compounds of the tubal fluid and summarize the factors that can dynamically change the early embryonic microenvironment. Because of ethical constraints; availability of sample material; and last but not least, the economic importance of fertility in livestock husbandry, a large part of the work on this topic has involved farm animals, and this review will largely focus on these species.

## 2. Origin and Renewal of the Tubal Fluid

**Figure 2:**
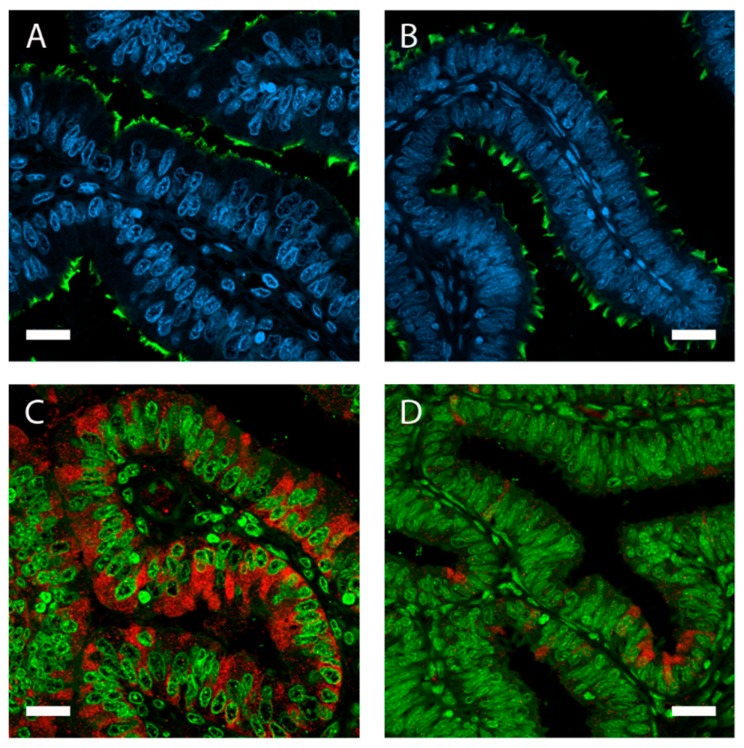
The oviduct lumen is lined by a simple, columnar-shaped epithelium containing non-ciliated and ciliated cells. Immunolocalization of acetylated tubulin (**A**,**B**; green) in porcine oviduct epithelium visualizes motile cilia bordering the lumen. Epithelial height, cell volume, and protein expression is influenced by estrous cycle stages. Oviduct tissue from the ampulla in (**A**) follicular and (**B**) luteal phase. Higher expression of oviductin (oviduct-specific glycoprotein 1 (OVGP1); red) in follicular (**C**) compared with luteal (**D**) phase. Nuclei stained with 4′,6-diamidino-2-phénylindole (DAPI) (**A**,**B**; blue) and SYBR Green (**C**,**D**; green). Magnification 400×, scale bars: 20 µm.

**Figure 3:**
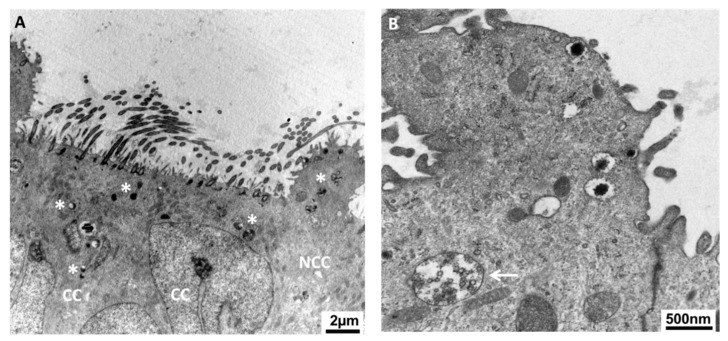
Both non-ciliated and ciliated cells are likely to participate in oviductal secretions. Transmission electronic microscopy reveals many secretory granules in both types of cells (**A**) as well as multivesicular bodies (MVB, **B**) close to the apical lumen of porcine oviduct epithelium. Asterisks indicate the secretory granules. The arrow points at the MVB. NCC: non-ciliated (secretory) cell; CC: ciliated cell.

The oviductal fluid (OF) is predominantly composed by the oviduct epithelium and consists of components that are either passively or actively transported over the epithelial barrier from the circulating blood or the interstitial tissue, or de novo secreted by the oviduct epithelial cells [8]. The oviduct lumen is lined by a simple, columnar-shaped epithelium containing both non-ciliated and ciliated cells (Figure 2). Although non-ciliated cells are also called ‘secretory non-ciliated cells’, it is most likely that both non-ciliated and ciliated cells participate in oviductal secretions (Figure 3).

### 2.1. Potential Participation of the Follicular Fluid in the Composition of the Tubal Fluid

Cyclic pick-up of COC(s) at one extremity of the oviduct, namely the infundibulum, raises the question of the participation of pre-ovulatory follicular fluid (FF) in the composition of OF. To answer that question, Brussow et al. compared progesterone concentrations in sow oviducts before and after ovulation, and after unilateral follicular fluid aspiration or oviduct ligation at infundibulum [9,10]. Progesterone levels in OF were around 1000 times lower than in FF and remained similarly low prior to and after ovulation, after follicle aspiration, or after oviduct ligation [10], indicating that the majority of FF did not flow down the oviduct after ovulation. Another study confirmed that only 0.5 ± 0.1% of the available progesterone in FF was present in the oviduct near the time of ovulation, and that this amount was decreased by 10–12-fold 4 h later [11]. Thus, in the porcine at least, FF seems to participate in OF composition only in negligible amounts. However, this is not proven yet in other species.

The contribution of FF components to oviductal secretions may be restricted to the passage of a few cumulus cells in a short time window, but yet with a significant impact on fertilization and embryo development. Finely-tuned interactions take place between cumulus cells and oviduct epithelial cells at ovulation [12], and cumulus cells are able to trigger specific expression patterns related to prostaglandin E_2_ (PGE_2_) and extracellular matrix in bovine oviductal cells in vitro [13]. Extracellular vesicles from the preovulatory follicle may also enter the oviduct at ovulation. In cattle, EVs from FF were reported to be internalized by developing embryos in vitro and to modulate metabolic and developmental-related genes as well as miRNA and global DNA methylation [14].

### 2.2. Production Rate and Renewal of Oviductal Secretions

The oviductal lumen is a dynamic milieu in which oviductal secretions are constantly renewed. In cyclic cows, the mean OF secretion rate measured using chronic oviduct cannulation was 0.2 mL per day during the luteal phase and 2.0 mL per day during estrus [15]. The quantity of OF recorded per day in pony mares was much higher, ranging from 0.8 to 3.5 mL during the luteal phase and from 3.2 to 6.4 mL during standing estrus [16]. Although valuable information can be obtained using in vivo cannulation of the oviduct, a long-term ligation certainly induces inflammatory responses in the tissue that can lead to artefacts in the collected fluid volume and composition [17]. A short-term collection during a 3 h anesthesia in heifers allowed measurement of a secretion rate of around 3 µL per minute on day 3 of the estrous cycle [18], corresponding to 4.3 mL per day. Taking into account the almost virtual lumen of the oviduct, such secretion rates indicate a continuous renewal of the oviductal microenvironment, particularly in the periovulatory period.

The mechanical flow generated by the constant renewal of oviductal secretions may be important for embryo development in vivo. A microfluidic ‘oviduct-on-a-chip’ platform, which provided a constant flow rate of medium on embryos, was reported to support more physiological (in vivo-like) zygote genetic reprogramming than conventional IVF in cattle [19].

The renewal of oviductal secretions also relies on the self-regeneration of the epithelial lining of the oviduct. Little is known about the mechanisms governing the homoeostasis and regeneration of the oviductal epithelium. The oviduct epithelial cells have been shown to proliferate mostly during the estrogen-dominant preovulatory phase of the cycle, while apoptotic cells were typically observed during the luteal phase in various mammals [20,21,22]. Therefore, the oviduct seems to have the regenerative potential to remodel itself across the estrous cycle. It was reported that especially the non-ciliated cells of the oviduct epithelium proliferate, whereas only a few or no dividing ciliated cells were observed, depending on the species [20,23]. It is likely that, under the influence of hormonal changes during the periovulatory stage, that is, decline of progesterone coupled with peak of estrogen, non-ciliated secretory cells self-renew and give rise to cells that terminally differentiate into ciliated cells [20,23].

## 3. Molecular Components of the Tubal Fluid

A complex mixture of molecules, from small metabolites and low molecular weight hormones to lipids, proteins, and high molecular weight glycoproteins, has been identified in oviductal secretions. This review focuses on recent data on this topic, obtained in particular thanks to mass spectrometry techniques.

### 3.1. Small Metabolites and Carbohydrates

Amino acids in OF may serve the embryo as precursors for proteins and nucleic acids, but also as energy source, signaling molecules, and osmolytes. All amino acids were identified in the mammalian OF, among which alanine, glycine, glutamine, and lysine were predominant and generally more abundant than in the circulating serum [24,25,26]. A high variability exists in the concentrations of metabolites measured in different studies for the same species [24,25,26]. Beyond the difference in quantification methods that were used, this variability may be because of the different methods of collection, which included ex vivo aspiration of OF from post-mortem genital tracts, chronic or acute cannulation of oviducts in situ, and conditioned media by oviduct epithelial cells in vitro [17].

To produce energy, the early embryo uses mostly oxidative metabolism (from lactate and pyruvate), and then progressively switches to a glycolytic metabolism as it leaves the oviduct [27]. Lactate is present in OF at much higher concentrations than in the serum and uterine fluid, whereas glucose is globally less present [25,28], in line with the metabolic needs of the early embryo. The synthetic oviductal fluid (SOF) medium was originally based on the concentrations of glucose, lactate, and pyruvate measured in the ovine OF [29] and, up to now, most culture media for embryos contain only these three energy substrates. However, a recent metabolomic analysis of the bovine OF reported a high number of additional energy substrates, including myoinositol, malonate, succinate, and glucose-1-phophate [26], that could be used to design more appropriate embryo culture media.

### 3.2. Lipids

Although carbohydrates have long been considered as the main energy source for embryos, lipids accumulate in both the oocyte and embryo and can provide a potential source of energy in addition to key roles in cell proliferation and differentiation. The embryo lipid reserve may be of particular importance in species with long preimplantation period such as pigs, cattle, dogs, and horses. Oviductal secretions contain a variety of lipids, including cholesterol, triglycerids, and fatty acids [30]. The OF contains also L-carnitine, which is required for the beta-oxydation of these lipids, while albumin as well as high- (HDLs) and low-density lipoproteins (LDLs) play the role of lipid carriers in OF [25,31]. Furthermore, a complex mixture of glycerophospholipids and sphingolipids, which are typical membrane lipids implied in many cell signaling pathways, was recently identified by mass spectrometry in the oviductal tissues and secretions in cattle [31,32]. Microvesicles and exosomes, which are typically rich in phospholipids similar in composition to those in cell membranes [33], are likely to contribute to the accumulation of phospholipids in OF.

Given the high abundance and diversity of lipids in OF, one can speculate about a role in embryonic development. However, current data on the effects of oviductal lipids on embryo development are scarce. At the expected day of embryo presence in the oviduct (day 4 after ovulation), distinct phospholipid profiles were evidenced between oviducts from beef females with contrasted embryo receptivity [32]. Furthermore, the addition of progesterone at intra-oviductal concentrations during in vitro culture changed the phospholipid composition of bovine embryos with a parallel improvement of their survival rate after vitrification [34].

### 3.3. Proteins

Recently, our knowledge on the proteomic content of OF in mammals has greatly increased thanks to mass spectrometry. Proteomes of the tubal fluid have been obtained in pigs [35,36], dogs [37], sheep [38], cattle [39,40,41], horse [42], rabbit [43], and human [44]. The most abundant proteins in oviductal secretions include serum albumin, oviductin (OVGP1), heat shock proteins (HSP90B1, HSP90AB1, HSP90AA1, HSPA8, HSPA1B, HSPA5), annexins (ANXA1, ANXA4), tubulin subunits (TUBB5, TUBA4A), complement C3 (C3), and myosins (MYH9, MYH14) [38,39,40]. A high proportion of metabolic regulators, enzymes, growth factors, and extracellular matrix components is also found in the oviductal fluid. The origin of proteins found in oviductal secretions is controversially debated. Among proteins identified in OF, a majority was classified as cytoplasmic proteins, whereas those possessing a signal peptide sequence, and can thus be classified as classically secreted, generally make up only a small fraction of the abundant proteins (13–27%) [38,39,41,43]. Lectin-based affinity enrichment of rabbit OF proteins increased the proportion of classically secreted proteins (mostly glycoproteins) to 63% [43]. It is unclear how intracytoplasmic proteins are exported in high proportions in oviductal secretions. The OF is classically collected by flushing or scraping the oviducts before centrifugation to eliminate cells and cell debris. This may lead to proteins released into the OF owing to unwitting cell damage. Alternatively, cytoplasmic proteins detected in the lumen may derive from the apocrine secretory pathway or be secreted via non-classical mechanisms [41,43], as known for annexins (ANXAs), heat shock protein 70 (HSP70), and interleukin 1-beta (IL-1β). Additionally, a majority of proteins identified in OF was also found in EV fractions isolated from the OF of cattle [45] and cat [46]. This is the case for OVGP1, heat shock proteins (HSPA8, HSP70), annexins (ANXA1-5), and several intracytoplasmic enzymes (e.g., fatty acid synthase (FASN) and glycogen phosphorylase (PYG)), among others. Thus, it is likely that a large part of proteins in the OF are actively released from oviduct epithelial cells as EV cargos.

Functional analysis of OF proteins revealed catalytic activity and binding as the main molecular functions [39,40,41,42]. In addition, a large proportion of highly abundant proteins in OF are involved in immune homeostasis (heat shock proteins, complement C3, alpha-2-macroglobulin, galectins, translation elongation factors, and cytokines) [38,41,43]. The oviduct hosts and provides for allogeneic spermatozoa and semi-allogeneic embryos, but at the same time, needs to keep up an adequate defense against pathogens, which is a challenging task from an immunological perspective. However, the specific roles played by oviductal HSPs and other secreted proteins related to the immune system in the oviduct are not well understood. In cattle, HSP90B1, HSP27, and GRP78 (also called HSPA5), among other proteins, were identified as interacting with spermatozoa [47]. From in vitro studies, it is known that oviductin, osteopontin, and complement C3 interact with the early embryo and support its development [48,49]. Recently, using mass spectrometry, more than 50 OF proteins, including oviductin, several annexins, alpha-2-macroglobulin, and galectin-3, were shown to interact with the cattle embryo [50]. Interestingly, a majority of these embryo-interacting proteins was also identified in bovine and feline oviductal exosomes [45,46], supporting the idea of an embryo–maternal dialog mediated by EVs in the oviduct.

### 3.4. Hormones

Hormones of low molecular weight (~300 Da), such as steroids and prostaglandins produced at high levels by ovarian follicles and corpora lutea, can reach the tubal fluid locally by vascular counter-transfer from the ovarian vein to the oviduct branch of the ovarian artery [51]. A local diffusion of hormones from the lymphatic network of the mesovarium (surrounding the oviduct) to the oviduct lumen may also be possible. The ovarian steroid hormones progesterone and estradiol are always detected in oviductal secretions, but their concentrations vary greatly between species. In rabbits during estrus, oviductal progesterone and estradiol were measured at concentrations more or less similar to those measured in the circulating serum (0.5 ng/mL of progesterone and 50–120 pg/mL of estradiol) [52]. By contrast, in pigs, cows, and mares, recent studies indicate that progesterone and estradiol are present in tubal fluid at much higher concentrations than those observed in the serum [39,53,54]. The fold-changes between OF and serum are particularly high in the oviducts ipsilateral to the ovulation side in cows and mares, in which progesterone and estradiol reach 10 to 50 times higher levels in the tubal fluid than in the circulating blood [39,54]. Steroid hormones other than progesterone and estradiol are also present in tubal fluid. Using a mass spectrometry approach, testosterone, estrone, cortisol, cortisone, and many metabolites of progesterone were detected in OF of cyclic cows throughout the estrous cycle [55]. Recent data in the equine indicate that the oviduct may be steroidogenic as enzymes such as StAR, cytochrome p450scc, and aromatase were immunodetected in oviduct epithelial cells [54]. This could explain the locally high intra-oviductal concentrations of progesterone measured after ovulation (up to 100 ng/mL) in this species [54].

Prostaglandins (PG) are also present in oviductal secretions. Both PGE_2_ and PGF_2α_ were reported in tubal fluid and in culture media conditioned by oviductal explants or epithelial cells in pigs, horse, and cattle [56,57,58]. The expression of numerous enzymes involved in prostaglandin synthesis has also been reported in the oviduct of various mammals [56,57,59]. Prostaglandins, together with steroid hormones, may participate in the optimal transport of gametes and embryo through their modulating effects on both the muscular contractility and ciliary beat frequency in the oviduct (for review, see the work of [60]).

There is little information on the presence of pituitary and chorionic gonadotrophins in oviductal secretions in mammals. However, the presence of human chorionic gonadotropin (hCG) was reported in oviducts from women during the menstrual cycle and in the post-partum period [61]. Moreover, the expression of receptors for luteinizing hormone chorionic gonadotropin (LHCG) and follicle stimulating hormone (FSH) was reported in the oviduct epithelium of several species including mouse [62], human [63], and pig [64,65]. The potential role of gonadotrophins in the oviduct is currently unknown, but treatments used for estrus synchronization, which typically use gonadotropins, may alter the oviduct microenvironment (see below).

## 4. Factors and Processes Regulating Oviductal Secretions

Various physiological factors have been identified as modulators of oviductal secretions. In addition, current reproduction management practices such as hormonal synchronization of estrus and insemination, as well as the metabolic imbalance and heat stress induced by intensive milk production, are susceptible to change the oviductal microenvironment. Figure 1 illustrates the local physiological processes influencing oviductal secretions. Table 1 summarizes main factors of regulation identified in vivo and their effects on oviduct epithelial cell (OEC) and OF molecular compounds.

### 4.1. Ovulation

Monovulatory species are valuable models to study the influence of ovulation and proximity of the corpus luteum on oviductal secretions. The question is particularly relevant just after ovulation, at the time of fertilization and embryo development. On day 3 following induced estrus in heifers, oviduct epithelial cells from the ipsilateral isthmus, where the embryo is expected to be located at this time, displayed 1927 differentially expressed genes (DEGs) compared with the contralateral oviduct [66], indicating that the endocrine signals associated with the ovulatory process and early corpus luteum had a significant impact on oviduct gene expression. However, the regulatory effect of the proximity of the corpus luteum was less pronounced in the ampulla than in the isthmus, as only 60 DEGs were identified between ipsilateral and contralateral ampullae [66].

The effect of ovulation on the oviductal proteome is also significant. In cyclic cows, 115 out of 482 (24%) proteins identified in OF in the post-ovulatory period differed in abundance between ipsilateral and contralateral sides relative to ovulation, and 17% differed between the pre-ovulatory and the post-ovulatory stages in ipsilateral OF [39]. Among the proteins upregulated in OF after ovulation, most were involved in the immune system and response to stress [39]. To a lesser degree, the comparison between ipsilateral and contralateral OF from cyclic mares evidenced only seven proteins differentially abundant [42]. The effect of the side of ovulation on protein abundance in OF may be related to important differences in the concentrations of progesterone and estradiol between ipsilateral and contralateral oviducts in monovulatory species [54,55]. Nevertheless, no significant differences have been detected for the relative levels of most OF metabolites and phospholipids between ipsilateral and contralateral oviducts in cattle [24,26,28,31].

### 4.2. Insemination and Gametes

In vivo, embryo development takes place in a microenvironment in which gamete transport, sperm capacitation, and fertilization took place a few minutes to hours previously (Figure 1). In pigs, there is evidence that the passage of hundreds to thousands of spermatozoa within the oviduct lumen alter the pattern of RNA synthesis and protein secretion in the epithelium [35,67]. The most secreted proteins changed by the presence of sperm cells were regulators of protein folding and stability such as protein chaperones [35,36]. The oviduct also seems able to sense subtle differences between populations of sperm cells, as differential transcriptomic signatures were evidenced between oviducts inseminated with X- and Y-bearing spermatozoa in gilts [67]. In rabbits, global alterations caused by insemination in the OF proteome were recently analyzed by mass spectrometry after lectin-based enrichment in glycoproteins [43]. The relative abundance of proteins markedly changed 4 and 8 h after insemination compared with both the time-matched controls and non-inseminated females [43]. These changes were region-specific as abundance of 11 proteins was specifically regulated in the isthmus, while 23 were modified only in the ampulla region 8 h after insemination. Functional enrichment analysis of proteins that were detected with an increased abundance upon insemination in both the ampulla and isthmus revealed response to stress, response to external stimulus, immune response, and antimicrobial humoral response as the main biological processes [43].

Spermatozoa, as alien cells for the maternal host, are expected to be rapidly eliminated from the oviduct. By contrast, spermatozoa able to pass through the utero–tubal junction and reach the isthmus can be stored on site for hours to days in close contact with the oviduct epithelial cells before being released for fertilization [68]. Thus, spermatozoa seem able to markedly modulate the immunotolerance of the oviduct. The bovine oviduct harbors two types of leukocytes (lymphocytes and neutrophils) and, under physiological conditions, neutrophils are present in OF before ovulation [69]. Interestingly, both the LH surge and the binding of spermatozoa to bovine oviduct epithelium have been shown to stimulate epithelial cells to secrete PGE_2_, which, in turn, suppressed phagocytosis of sperm cells and the synthesis of pro-inflammatory cytokines by neutrophils in vitro [69,70]. In the time window of ovulation, follicular and oviductal fluids seem to have opposite effects on the oviduct immunotolerance. The follicular fluid collected from buffalo pre-ovulatory follicles enhanced sperm phagocytosis by neutrophils through the formation of neutrophil extracellular traps (NETs) and H_2_O_2_ formation, whereas OF inhibited this activity in vitro [71]. Thus, the oviductal milieu seems to minimize the inflammatory effect of the ovulatory process, and may thereby promote sperm survival.

Although in vivo data are scarce, the oocyte–cumulus complex also seems to be able to modulate the protein secretion of the oviduct epithelium [35,36]. Some of the oocyte-modulated proteins identified in pigs were not identified after co-incubation with spermatozoa, supporting the notion of a specific effect of the oocyte on the oviduct [35,36]. Furthermore, the cumulus cells around the oocyte may themselves interact with the oviduct epithelium or mediate and amplify the signals emitted by the oocyte.

### 4.3. Pregnancy and Embryo

Several studies support the idea that the embryo is able to ‘drive’ its own transport through the oviduct. The recovery rate of bovine embryos in the uterus after tubal transfer in synchronized heifers differs depending on the morphological and structural properties of the embryos [72]. There is some evidence that the embryo plays an active role in opening the utero–tubal junction for its route to the uterus. In the equine species, non-fertilized oocytes and early arrested embryos remain trapped within the oviduct, whereas embryos beyond the 16-cell stage can usually be recovered from the uterus after day 6 post-insemination [73]. Prostaglandin E2 seems to be the key molecule allowing the developing equine conceptus to open the ‘door’ of the utero–tubal junction [74]. Using a videomicroscopic system on bovine oviducts ex vivo, Kolle et al. evidenced local downregulation of transport speed at the specific site of embryo presence compared with areas of ampulla and isthmus without an embryo [12], supporting the idea that the embryo is able to modulate the oviductal ciliary beating.

Although there is evidence for specific effects of oviductal secretions on embryo development and quality [2,75,76,77], the impact of the early embryo on the oviduct epithelial cell gene expression is less documented. In mice, it was already shown 18 years ago that the presence of early embryos in the oviduct is able to upregulate the expression of specific genes compared with non-fertilized oocytes [78]. In pigs, the expression of *TICAM2*, an immune-related gene, was shown to be regulated in the oviduct epithelium by the presence of embryos, and this regulation was dependent on the embryo developmental stage [79]. In vitro, there is evidence that bovine embryos co-cultured with bovine oviduct epithelial cells (BOEC) trigger specific changes in BOEC gene expression as early as the two-cell stage [80,81]. Of note, many transcriptomic changes induced by cattle and pig embryos in the oviduct were related to immune functions and the interferon–tau signaling pathway [6,81].

However, whether a single embryo is able to alter the oviduct transcriptome in vivo has still not been conclusively clarified in monovulatory species. In heifers, the presence of a single eight-cell embryo did not have any detectable effect on the transcriptome of epithelial cells in the isthmus, where the embryo is located at day 3 post-estrus [82], pointing out that the data reported from litter-bearing species are probably cumulative effects from a group of embryos. In accordance, the endoscopic transfer of 50 in vitro produced bovine zygotes on day 1.5 post-estrus in heifers led to significant changes in the gene expression of the isthmus on day 3 post-estrus [82]. In order to avoid a dilution of an embryonic effect in a physiological situation, Rodriguez-Alonso et al. [83] focused on 2 cm oviduct sections in which the cattle embryo was retrieved on day 2.5 after insemination and made comparisons on candidate gene expression with adjacent sections in the same oviduct or with the same section in the contralateral oviduct. However, no site-specific effect of the cattle embryo could be evidenced with this method.

By contrast, the comparison between ipsilateral (containing the embryo) and contralateral oviducts within pregnant mares on day 4 post-ovulation indicated 164 DEGs in oviduct epithelial cells [84] and 13 differentially abundant proteins in OF [42]. The cells were collected at the ampulla–isthmus junction, where the embryo stays during its development, to avoid a dilution of the embryonic effect. Nevertheless, the effect of the embryo may be confounded with the endocrine-related effect of the proximity of the corpus luteum described previously. By comparing ipsilateral oviducts from pregnant and cyclic mares on day 4 post-ovulation, 361 RNAs in oviductal cells and 19 proteins in OF were found to be differentially abundant [42,84], indicating the potential impact of the early embryo on oviductal secretions. However, up to now, only in vitro models make it possible to distinguish the specific effect of the embryo from those of spermatozoa previously present in the same site as well as from the global effect of pregnancy on oviductal secretions.

### 4.4. Sex Steroid Hormones

In cattle, pig, and horse, concentrations of progesterone in the circulating blood are generally under the detection level during the early post-ovulation period. Nonetheless, in those species, the shift from an estrogen-dominant to a progesterone-dominant hormonal environment is well detectable in the oviductal tissue and tubal fluid, which display high detectable levels of steroid hormones in the periovulatory period [53,54,55,85]. Therefore, the early embryo is exposed to relatively high levels of steroid hormones in the oviduct.

**Figure 4:**
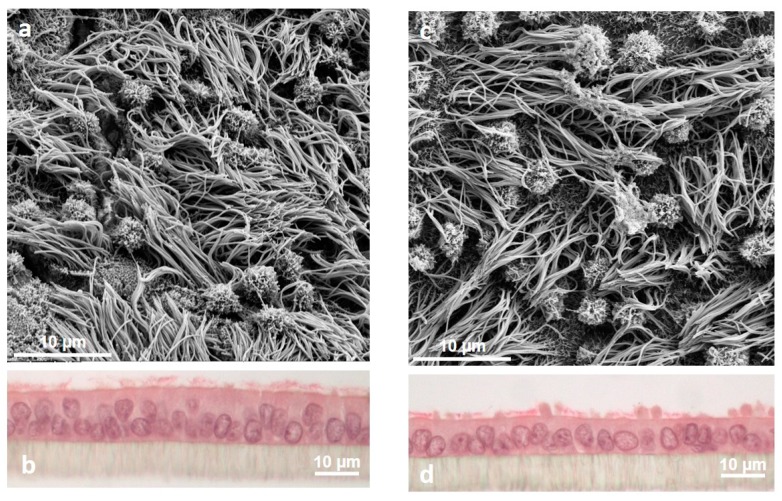
Morphological changes of porcine oviduct epithelial cells under the influence of sex steroid hormones during simulated estrous cycle in vitro. Scanning electron microscopy showed the apical ultrastructure in simulated follicular (**a**) and luteal phase (**c**), respectively; magnification 5000×. (**b**) and (**d**): Hematoxylin eosin staining; magnification 400×.

Sex steroid hormones are definitely major players of oviductal morphology and physiology (for review, see the work of [86]). Estrogens induce hypertrophy and an increase in cell height of the oviduct epithelial cells, while progesterone causes atrophy and reduced cell secretion (Figure 4) [8]. During the first six days after ovulation and under the influence of increasing concentrations of progesterone, the secretion rate of OF shows a tendency to decline in cattle [18,28]. Also, changes in the concentrations of various compounds in bovine oviductal secretions have been reported in parallel with the inversion of the local or systemic estradiol/progesterone ratio around the time of ovulation, including amino acids [18,24,26], energy substrates [18,26,28], prostaglandins [85], phospholipids [31], and proteins [39].

Estrogens have been associated with an increase in oviductin secretion in the oviduct of cows, sheep, pig, and human (Figure 2) [87]. Furthermore, estrogen-response, but not progesterone-response elements were found in the upstream regulatory region of the gene coding for oviductin [88]. However, recent in vitro studies demonstrated that progesterone has a major influence on the abundance of OVGP1 mRNA even in the absence of estradiol [89]. Progesterone is also likely to regulate the secretion of specific proteins to the oviductal lumen. The treatment of canine females with the progesterone receptor antagonist aglepristone at day 4 post-ovulation induced changes in the abundance of 23% (79/343) of identified proteins in oviductal secretions [37]. In the bovine OF, local concentrations of progesterone correlated with the abundance of some proteins including phosphatidylethanolamine-binding protein 1 (PEBP1) and peroxiredoxin-2 (PRDX2) [39]. However, the impact of progesterone on other OF compounds lacks evidence. In heifers supplemented with systemic progesterone on day 3 post-estrus, the most marked effects of progesterone were on amino acid concentrations in oviductal secretions, with a maximum positive effect on glycine, whereas ions did not notably change in concentrations and there was no effect on energy substrates [18].

The mechanisms by which steroid hormones regulate oviductal secretions are not fully understood. Both classical nuclear receptors and non-classical membrane receptors for estrogens and progesterone are present in the oviduct epithelium [90,91]. Ovarian steroid hormones may regulate OF secretion rate and composition through several mechanisms, including changes in cell morphology, modulation of the blood flow to the oviduct, and regulation of the transport of ions and glycoproteins through the oviduct epithelium, leading to changes in intraluminal osmolarity [8,17].

### 4.5. Treatments of Estrus Synchronization and Superovulation

For technical and economic reasons, treatments of estrous synchronization using progesterone agonists and equine chorionic gonadotropin (eCG) are typically used before insemination in ewes and also frequently used in sows and cows. Protocols with eCG, FSH, and/or hCG are also successful methods for superovulation before embryo collection in pig and cattle. Exogenous chorionic gonadotropins act through LHCG and FSH receptors, and their elimination half-life exceeds those of pituitary gonadotropins in various species [92]. Although the expression of receptors for LHCG and FSH receptors has been demonstrated in oviducts of several mammalian species [63,64,65], the potential effects of superovulation treatment on oviduct physiology are not well documented. In gilts, it was shown that synchronization treatments with progesterone and eCG/hCG can alter the epithelium height and the proportions of secretory cells in the oviduct epithelium [93]. Stimulation with hCG/eCG before insemination in gilts decreased the synthesis of PGE_2_ and increased those of PGF_2α_ in the oviductal ampullas and isthmus three days post-insemination [59]. In Nelore cows, superovulation treatment using FSH and eCG induced site-specific changes in the expression of receptors for prostaglandins (EP2 and EP4) and angiotensin II (AGTR2) in oviduct epithelial cells [94]. In Buffalo cows treated with repeated doses of FSH for superovulation, a decreased expression of progesterone receptor (PGR) and estrogen receptor 1 (ESR1), as well as vascular endothelial growth factor (VEGF) and its receptor vascular endothelial growth factor receptor 1 (FLT1), was observed in the oviduct [95]. These changes may account for the failure of oocyte capture by the oviduct and subsequent low embryo recovery rate observed in superovulated buffaloes [96]. These various data suggest that treatments with exogenous gonadotropins alter prostaglandin and steroid signaling pathways that may in turn disturb oviductal secretions and muscular contractions for gametes/embryo transport.

One study compared the effect of the stage of cycle (estrus vs. luteal phase) and of the type of cycle (synchronized vs. spontaneous) on the proteome of the oviductal fluid in ewes [38]. The ratios of differentially abundant proteins between estrous and luteal phases were similar between spontaneous and synchronized estrus. However, only 42% (34/81) of differentially abundant proteins were shared between both spontaneous and synchronized estrus [38], indicating that the hormonal treatment induced specific changes in oviductal secretions along the estrous cycle.

### 4.6. Metabolism and Energy Balance

The oviduct can be considered as a ‘supply line’ between the mother diet at one end and the conceptus at the other, with a potential long-term effect on pregnancy and newborn health [17]. As the early embryo is highly sensitive to metabolic perturbations in its environment, the oviduct is expected to maintain an optimal environment with homeostatic mechanisms. However, there is evidence that metabolic perturbations encountered by the mother can alter this buffering function of the oviduct [97]. In goats, a modification of the nutritional balance in the diet altered the expression of proteins participating in apoptosis, antioxidant, and immunological activities in the periovulatory oviduct—functions important for embryo development [98].

Intensive selection for milk yield in dairy cows has led to metabolic perturbations associated with postpartum negative energy balance and decreased embryo survival. Studies in which bovine embryos were transferred into the oviduct of animals of different metabolic status indicated that the oviductal microenvironement is compromised by lactation [99,100]. Accordingly, two weeks postpartum in lactating cows, when the energy balance is already negative, specific changes in insulin growth factor-binding protein (IGF-BP) expression were reported in the oviduct [101]. Recent data indicate that, at the expected time of conception (60 days postpartum), the transcriptome of isthmus epithelial cells and proteomic composition of oviductal fluid differed between age-paired Holstein lactating and non-lactating cows [66,102]. The changes in gene and protein expression were moderate; only 15 DEGs and 12 differentially abundant proteins were identified between age-paired lactating and non-lactating cows [66,102]. Nevertheless, lactation strongly altered the effect of proximity to corpus luteum on oviduct gene expression. There were up to 14 times more DEGs between ipsilateral and contralateral oviducts in non-lactating than in lactating cows (139 vs. 10 DEGs in ampulla; 1275 vs. 331 DEGs in isthmus) [66], suggesting that the metabolic stress associated with lactation impaired the local regulatory signal emitted by the ipsilateral ovary.

Intensive energy demand is often manifested through elevated non-esterified fatty acids (NEFAs) in the circulating blood. In healthy cattle, levels of NEFAs in the tubal fluid mirror the concentrations measured in plasma [30], meaning that, in the case of lipolytic metabolism during the periconceptional period, the early embryo is likely exposed to elevated NEFAs. Elevated levels of NEFAs were shown to affect embryo DNA methylation and gene expression [103]. In addition, it has been demonstrated that elevated NEFAs affect the metabolism and barrier function of the oviduct epithelium in vitro [97], reinforcing the idea that the buffering function of the oviduct for the embryo can be compromised by maternal metabolic deflection.

### 4.7. Heat Stress

Global warming and intensive milk or meat production have reinforced the need to better understand the effect of heat stress on physiological functions of the body, including the female genital tract and possible impact on fertility. The embryo is particularly sensitive to heat stress at early stages of development [104] and an elevation in body temperature may be of particular importance during its oviductal stay. In pigs, rabbits, and human, some evidence suggests that a temperature gradient from the isthmus to the ampulla plays a role in sperm guidance to the oocyte and influences gene expression in the early embryo [27,105,106]. Therefore, a dysregulation of homeothermy in the oviduct may have a significant impact on fertility. Heat shock proteins (HSPs) are highly abundant in oviductal secretions of all mammals studied so far [38,39,40,41,42]. HSPs protect cells from various stresses including heat and reactive oxidants, but are also involved in intracellular protein transport and conformation. Bovine ampullar tissues retrieved during summer heat stress showed a parallel increased expression of PGE synthase 3 and HSP90AA1, suggesting an interference between HSPs and the prostaglandin pathway [57]. Bovine oviduct epithelial cells exposed to heat stress in vitro also showed increased secretion of PGE_2_ [57], an important factor for oviduct motility. In another study, long-term (168 h) exposure of bovine oviduct epithelial cells to high temperature (41 °C) increased the expression of HSP70 with no effect on cell viability [107], in accordance with the putative protective role of heat shock proteins. However, such elevated temperature decreased the cell expression of OVGP1 and prevented the embryos co-cultured with oviductal cells from developing beyond the four-cell stage [107]. Thus, the thermosensitivity of the oviduct and the protective role—or, conversely, the aggravating role—this organ could play on embryo development in the case of heat stress need further investigation.

## 5. Concluding Remarks and Perspectives

Under physiological conditions, the oviductal fluid is considered as the optimal milieu for the early developing embryo. Its basic composition with respect to ion content and energy substrates has been known for decades, and media that mimic this composition are successfully used for in vitro embryo production. However, it becomes more and more evident that the oviductal fluid is a highly dynamic microenvironment, which is regulated by a wide range of different effectors and influencing variables of which only a small number are currently known. The orchestrated action of systemic effectors (e.g., ovarian hormones) and local cell–cell interactions modulate oviductal secretions and immune responses, thus optimally adjusting the oviductal fluid composition to nurture and protect the embryo according to its changing developmental needs. Increasing incidence of metabolic disorders and infertility (with early embryonic loss as one of the major symptoms) in both human and livestock species with a simultaneous increase in use of reproductive biotechnologies worldwide calls for a better understanding of the dynamic early embryonic environment and its impact on fertility, embryo development, and offspring health.

New experimental approaches and model systems will be needed in the future to support these ambitious scientific endeavors. Oviductal fluid formation and its regulation is challenging to study in vivo because of ethical and technical constraints. Therefore, interdisciplinary efforts between reproductive medicine/biology, material sciences, and biomedical engineering are currently being undertaken to advance informative in vitro models. Three-dimensional, compartmentalized culture systems, which allow fluid formation across the polarized oviductal epithelial barrier and embryo co-culture, as well as perfused culture systems or microfluidic devices and combinations thereof, are emerging. With these models, various dynamic effectors and their influence on the composition of the early embryonic environment can be systematically tested.

In general, a much more precise characterization of the species-specific spatial and temporal changes in oviductal fluid composition during the time of embryo transit through the oviduct is needed. Beyond that, arising research areas are the existence and biological relevance of local cell–cell interactions, which potentially influence the OF micro-composition at the site of embryo presence. Finally, new knowledge is also needed on the adaptive competence and plasticity of embryos in response to (physiological and pathological) changes in the early embryonic environment and their implications for the embryo’s later development.

## Figures and Tables

**Table 1:** Summary of factors regulating oviductal secretions in vivo. OF, oviductal fluid; OEC, oviductal epithelial cell; DEGs, differentially expressed genes; TICAM2, Toll-like receptor adaptor molecule 2; hCG, human chorionic gonadotropin; eCG, equine CG; FSH, follicle stimulating hormone; EP2,4, prostaglandin E2 receptor 2,4; AGTR2, angiotensin II receptor type 2; PGR, progesterone receptor; ESR1, estrogen receptor 1; VEGF, vascular endothelial growth factor; FLT1, vascular endothelial growth factor receptor 1;; IGFBP, insulin growth factor binding protein.

Factor Studied	OF or OEC Component	Species	Main Result	Ref.
Ovulation	OF proteins	Cattle	Comparison between ipsilateral and contralateral oviducts identified up to 115 differentially abundant proteins across the estrous cycle	[39]
Ovulation	OF proteins	Horse	Seven proteins were differentially abundant between ipsilateral and contralateral oviducts in non-pregnant mares	[42]
Ovulation	OF steroid hormones	Cattle	Concentrations of OF progesterone and progesterone metabolites differed between ipsilateral and contralateral oviducts across the estrous cycle	[55]
Ovulation	OF steroid hormones	Horse	Concentrations of OF progesterone differed between ipsilateral and contralateral oviducts in the post-ovulatory period	[54]
Proximity of the corpus luteum	OEC gene expression	Cattle	Irrespective of the metabolic status of females, comparison between ipsilateral and contralateral oviducts identified 192 and 2583 DEGs in the ampulla and isthmus, respectively, on day 3 post-estrus	[107]
Proximity of the corpus luteum	OEC gene expression	Horse	Comparison between ipsilateral and contralateral oviducts indicated 164 DEGs in pregnant mares and 77 DEGs in cyclic mares	[84]
Ovulation and Insemination	OF proteins	Pig	Spermatozoa and oocyte–cumulus complexes altered the oviductal secretory proteome 24 h after ovulation and insemination	[35]
Insemination	OF proteins	Rabbit	Secreted OF proteins changed 4 and 8 h after insemination with region-specific alterations	[43]
Sex-sorted spermatozoa	OEC gene expression	Pig	Differentially expressed genes were identified in OECs in the presence of Y-chromosome bearing spermatozoa compared with X-chromosome bearing spermatozoa	[66]
Presence of embryos	OEC gene expression	Mouse	The expression of specific genes was upregulated in OECs in the presence of early embryos compared with non-fertilized oocytes	[78]
Presence of embryos	OEC gene expression	Pig	The expression of *TICAM2* was upregulated in the oviduct epithelium by the presence of embryos	[79]
Presence of one and multiple embryos	OEC gene expression	Cattle	The presence of multiple embryos in the oviduct resulted in the detection of DEGs in the isthmus of beef heifers on day 3 post-estrus; no DEGs could be detected in the presence of a single eight-cell embryo	[82]
Presence of one embryo	OEC ciliary beating	Cattle	A local downregulation of particle transport speed was evidenced in the site of the embryo in oviduct sections ex vivo	[12]
Pregnancy	OF proteins	Horse	The presence of an embryo in the ipsilateral OF of pregnant mares induced regulation of 13 proteins compared with the contralateral side, and of 19 proteins compared with the ipsilateral side of non-pregnant mares.	[42]
Pregnancy	OEC gene expression	Horse	Comparison between ipsilateral pregnant and non-pregnant oviducts identified 253 upregulated genes and 108 downregulated genes in OECs	[84]
Sex steroid hormones	Phospholipids	Cattle	Different phospholipid profiles were evidenced in oviducts from females with contrasted progesterone and estradiol levels during early diestrus	[32]
Stage of cycle	OF steroid hormones, proteins, metabolites, and lipids	Cattle	Comparison between four stages of the estrous cycle identified differentially abundant OF sex steroid hormones, proteins, amino acids, energy substrates, and phospholipids in both sides relative to ovulation	[26,31,39,58]
Stage of cycle	OF steroid hormones	Horse	Comparison between pre-ovulatory and post-ovulatory oviducts identified differential OF levels of progesterone in the side of ovulation	[54]
Progesterone	OF proteins	Dog	Treatment with the progesterone receptor antagonist aglepristone induced changes in the abundance of 79 OF proteins at day 4 post-ovulation	[37]
Progesterone	OF ions and metabolites	Cattle	Systemic supplementation with progesterone induced changes in OF amino acids, sulfate, and sodium	[18]
Superovulation treatment	Prostaglandin synthesis	Pig	Stimulation with hCG/eCG before insemination affected prostaglandin synthesis pathway on day 3 post-estrus in gilts	[59]
Superovulation treatment	OEC gene expression	Cattle	Superovulation treatment with FSH and eCG changed the expression of prostaglandin receptors EP2 and EP4 in the ampulla and infundibulum and of AGTR2 in the isthmus	[94]
Superovulation treatment	OEC gene expression	Cattle (Buffalo)	Superovulation treatment with FSH decreased the expression of steroid hormone receptors PGR and ESR1, VEGF, and its receptor FLT1	[95]
Estrus synchronization	OF proteins	Sheep	Proteins found differentially abundant between estrus and the luteal phase differed when comparing ewes in spontaneous cycles with those treated for estrus synchronization	[38]
Energy balance	OEC proteins	Goat	Comparison between four different diet groups identified seven differentially expressed proteins in ampullas of animals fed with 1.9 times live weight maintenance	[98]
Energy balance	OEC gene expression	Cattle	Negative energy balance was associated with changes in gene expression of IGFBP-2 and IGFBP-6 in the oviducts of lactating dairy cows	[101]
Energy balance	OEC gene expression	Cattle	Comparison between OECs from postpartum lactating and non-lactating dairy cows evidenced 15 DEGs in the isthmus and none in the ampulla	[66]
Energy balance	OF proteins	Cattle	Comparison between OF from postpartum lactating and non-lactating dairy cows evidenced 12 differentially abundant proteins	[102]

## Abbreviations

AGTR2	Angiotensin II receptor type 2
COC	Cumulus–oocyte complex
DAPI	4′,6-diamidino-2-phénylindole
DEGs	Differentially expressed genes
eCG	Equine chorionic gonadotropin
EP2,4	Prostaglandin E2 receptor 2,4
ESR1	Estrogen receptor 1
EVs	Extracellular vesicles
FF	Follicular fluid
FLT1	Vascular endothelial growth factor receptor 1
FSH	Follicle stimulating hormone
hCG	human chorionic gonadotropin
HSP	Heat shock protein
IVF	In vitro fertilization
LHCG	Luteinizing hormone chorionic gonadotropin
OEC	Oviductal epithelial cell
OF	Oviductal fluid
OVGP1	Oviduct-specific glycoprotein 1
PG	Prostaglandins
PGR	Progesterone receptor
SOF	Synthetic oviductal fluid
TICAM2	Toll-like receptor adaptor molecule 2
VEGF	Vascular endothelial growth factor
